# Bilirubin Oxidase from *Myrothecium verrucaria* Physically Absorbed on Graphite Electrodes. Insights into the Alternative Resting Form and the Sources of Activity Loss

**DOI:** 10.1371/journal.pone.0132181

**Published:** 2015-07-21

**Authors:** Federico Tasca, Diego Farias, Carmen Castro, Cristina Acuna-Rougier, Riccarda Antiochia

**Affiliations:** 1 Departamento de Química de los Materiales, Facultad de Química y Biología, Universidad de Santiago de Chile, Santiago, RM, Chile; 2 Facultad de Ciencias Naturales, Matemáticas y Medioambiente, Universidad Tecnológica Metropolitana, Santiago, RM, Chile; 3 Department of Chemistry and Drug Technologies, Sapienza University of Rome, Piazzale Aldo Moro 5, 00185, Rome, Italy; Wageningen University, NETHERLANDS

## Abstract

The oxygen reduction reaction is one of the most important chemical processes in energy converting systems and living organisms. Mediator-less, direct electro-catalytic reduction of oxygen to water was achieved on spectrographite electrodes modified by physical adsorption of bilirubin oxidases from *Myrothecium verrucaria*. The existence of an alternative resting form of the enzyme is validated. The effect on the catalytic cycle of temperature, pH and the presence of halogens in the buffer was investigated. Previous results on the electrochemistry of bilirubin oxidase and on the impact of the presence of halogens are reviewed and reinterpreted.

## Introduction

The oxygen reduction reaction (ORR) is one of the most important chemical processes in energy converting systems and living organisms [1,2]. Multicellular living organisms use oxygen as electron (e^−^) acceptor (e.g., the respiratory chain) [3]. In fuel cells chemical energy is converted into electrical energy through the ORR [1]. On the other hand, many fungi and bacteria use oxygen to form hydrogen peroxide to degrade wood and lignocelluloses and as a signaling molecule [3–5]. Insights into the ORR mechanisms would influence many aspects of our lives, from how we generate energy and fuels to medicine and biology [1,6].

Many studies have been conducted on the multicopper oxidases (MCOs) with the aim of gaining more insight into the ORR and how to reproduce this reaction with synthetic and biological materials [7]. For example, bilirubin oxidase (BOD) has been found to be the best catalyst for converting oxygen directly to water because of the very low overpotential necessary to catalyze the reaction [8], and a turnover rate of 0.7 O_2_ per Cu s^-1^, while for Pt nanoparticulate catalysts the turnover rate is three times lower at an overpotential of 350 mV [8,9]. Moreover MCOs play critical roles in Fe, Cu and O_2_ metabolism and are directly related to a range of genetic diseases (oculocutaneous albinism, aceruloplasminemia, etc.) and health issues (atherosclerosis, control of neurotransmitters, etc.) [10,11]. Understanding Cu biochemistry at the molecular level provides mechanisms to improve or inhibit these processes, and enable drug design and the development of fuel cells and implantable devices (i.e., biosensors, biofuel cells) [10,11].

BOD together with laccase, ascorbate oxidase, CotA, Fet3p, CueO, ceruloplasmin, etc., belong to the MCO family [7,11]. These enzymes have four copper atoms that are classified into three types according to their spectroscopic and magnetic properties: type I (T1), type II (T2) and type III (T3) Cu [7,11]. The MCO family can be separated into two types by substrate specificity. The first group catalyzes the outer sphere oxidation of small organic substrates and include the plant and fungal laccases and ascorbate oxidase, CotA, BOD, and phenoxazinone synthase [7,11]. The second group oxidizes metal ions at highly specific metal ion binding sites, and include Fet3p, CueO, and ceruloplasmin [7,11].

BOD was discovered in 1981 by Tanaka and Murao [12]. It catalyses the oxidation of bilirubin to biliverdin [12]. It has been used primarily in the determination of bilirubin in serum and thereby in the diagnosis of jaundice [13]. Here we will first describe the ORR mechanism with the latest details. Then we will describe the impact of temperature, pH, and the presence of chloride on the catalysis of ORR when BOD from *Myrothecium verrucaria* (*Mv*BOD) is immobilized on spectroscopic graphite electrodes (SPGE). Finally, we will link those experiments to previous studies and we will discuss the implications for the development of biocathodes.

## Materials and Methods

ABTS and dithionite were from Sigma-Aldrich (St. Louis, MO). All other products were analytical reagent grade and were used as received. *Myrothecium verrucaria* BOD Amano 3 was donated by Amano Enzyme Inc., U.S.A (http://www.amano-enzyme.co.jp/) and used as received. The purity of the provided power was 90% or higher. ABTS specific activity of BOD was measured by monitoring the increase in absorbance at 420 nm (ε = 36mM^-1^cm^-1^) over time with stirring, in 0.1 M sodium phosphate buffer and resulted to be 12 U/mg. One unit is defined as the amount of enzyme that oxidizes 1 μmol ABTS per minute. While a much higher activity (388 U/mg) has been reported for a purified preparation of *Mv*BOD [14] we used the enzyme as received. Spectrographic graphite electrodes (SPGE) were provided by Ringsdorff Werke GmbH, Bonn, Germany, (type RW001, 3.05 mm diameter and 13% porosity, http://www.sglcarbon.com). SPGE were prepared by wet polishing the end of a rod using waterproof emery paper. The electrodes were then rinsed with Milli-Q water and allowed to dry in air. Subsequently, 10 μL of enzyme solution (~0.2 mM) was placed on top of the polished rod and adsorption was allowed to occur. The electrodes were then stored overnight at 4°C under controlled humidity. After 24 h the electrode was rinsed with Milli-Q water, inserted in a Teflon holder, and used as working electrode. All spectroscopic data were obtained with BOD in 0.1 M sodium phosphate buffer, pH = 7, unless otherwise stated. The electrochemical experiments were carried out with a conventional three-electrode cell and a BAS-100 potentiostat (USA) or Autolab PGST30 potentiostat/galvanostat (Netherlands). Platinum spiral wire of 2 cm^2^ geometric area was used as the counter electrode, and a calomel saturated electrode (SCE) as the reference electrode. Electrolytic solutions were routinely deoxygenated with nitrogen gas. All potential values are given versus the normal hydrogen electrode (NHE). The current densities were calculated with respect to the geometric electrode area. All the experiments were repeated at least five times leading to similar results. However, there was some variation (up to 10 μA/cm^2^) in the absolute amount of current since the electrodes were polished and prepared manually. This error accounts for less than 5% of the maximum measured current (≈ 200 μA/cm^2^ at 0.4 V vs. NHE or ≈ 300 μA/cm^2^ at 0 V vs. NHE).

Reduction of as-isolated BOD was performed by adding small increments of a 0.1 M dithionite solution to argon purged (~4 h) enzyme, in nitrogen dry box. Prior to reoxidation, excess dithionite was removed from the reduced enzyme by buffer exchange into degassed sodium phosphate (0.1M) pH 7 buffer using Amicon Ultra centrifugal filters (10 kDa). Reoxidation was accomplished by addition of O_2_-saturated sodium phosphate (0.1M) pH 7 buffer. UV−visible (UV-vis) absorption spectra were obtained with an Agilent 8453 diode array spectrophotometer.

## Results and Discussion

**Fig 1:**
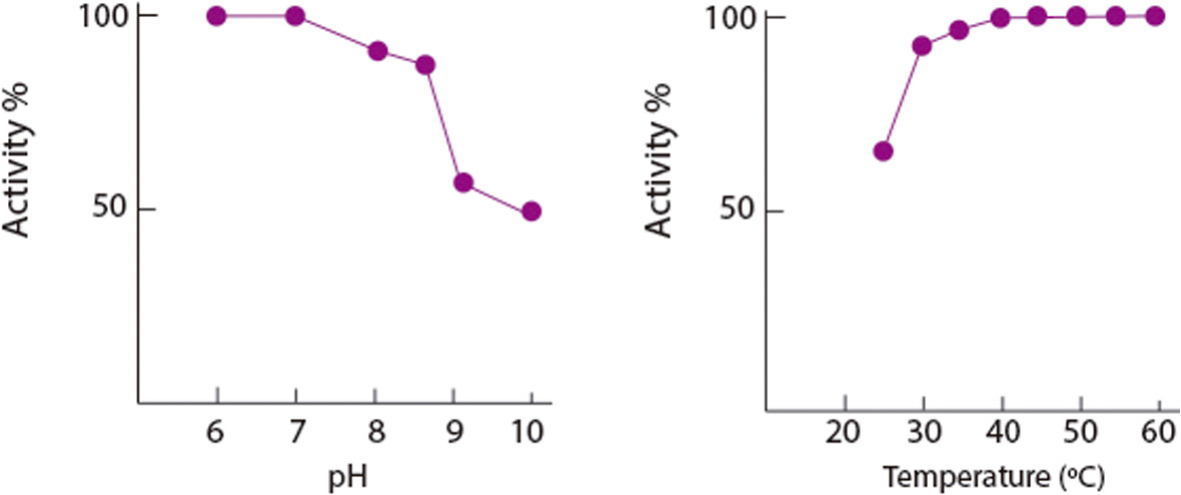
Activity profile at various temperatures and pH for *Mv*BOD physically adsorbed on SPGE. Sodium phosphate (0.1 M) buffer, pH 7. Electrode set at 600 mV vs. NHE.

Due to its use in the diagnostic field, BOD is commercially available and produced by Amano Enzymes Inc., among several companies. Unlike laccases, the most widely studied of the MCOs, BODs display higher activity and stability at neutral pH and high temperature (i.e. < 60°C) [15]. *Mv*BOD showed highest catalytic currents for temperatures of 30 to 60°C and pH in the range of 7 to 8.5, and loss of 50% of its activity at pH 9 (Fig 1). The activity appears to remain constant up to 60°C which is in agreement with the data reported by the manufacturer (http://www.amano-enzyme.co.jp/). Unfortunately due to the media we could not perform experiments at higher temperatures and we could not appreciate a decrease of the activity.

The onset potential of BOD activity was found to depend on pH (~30 mV/pH unit, E^0^ at pH 7 ~800 mV), with higher potentials found at low pH. It is generally accepted that MCO activity is inhibited at high pH, but elucidation of the inhibition mechanisms is still missing. Most probably, the electron transfer (ET) from T1 to the trinuclear site is affected. A problem of T1 would also account for the potential change of T1 at different pH values. Temperature and pH are extremely important factors when considering the design of biofuel cells because they allow various cell designs and various reactions at the anode.

**Fig 2:**
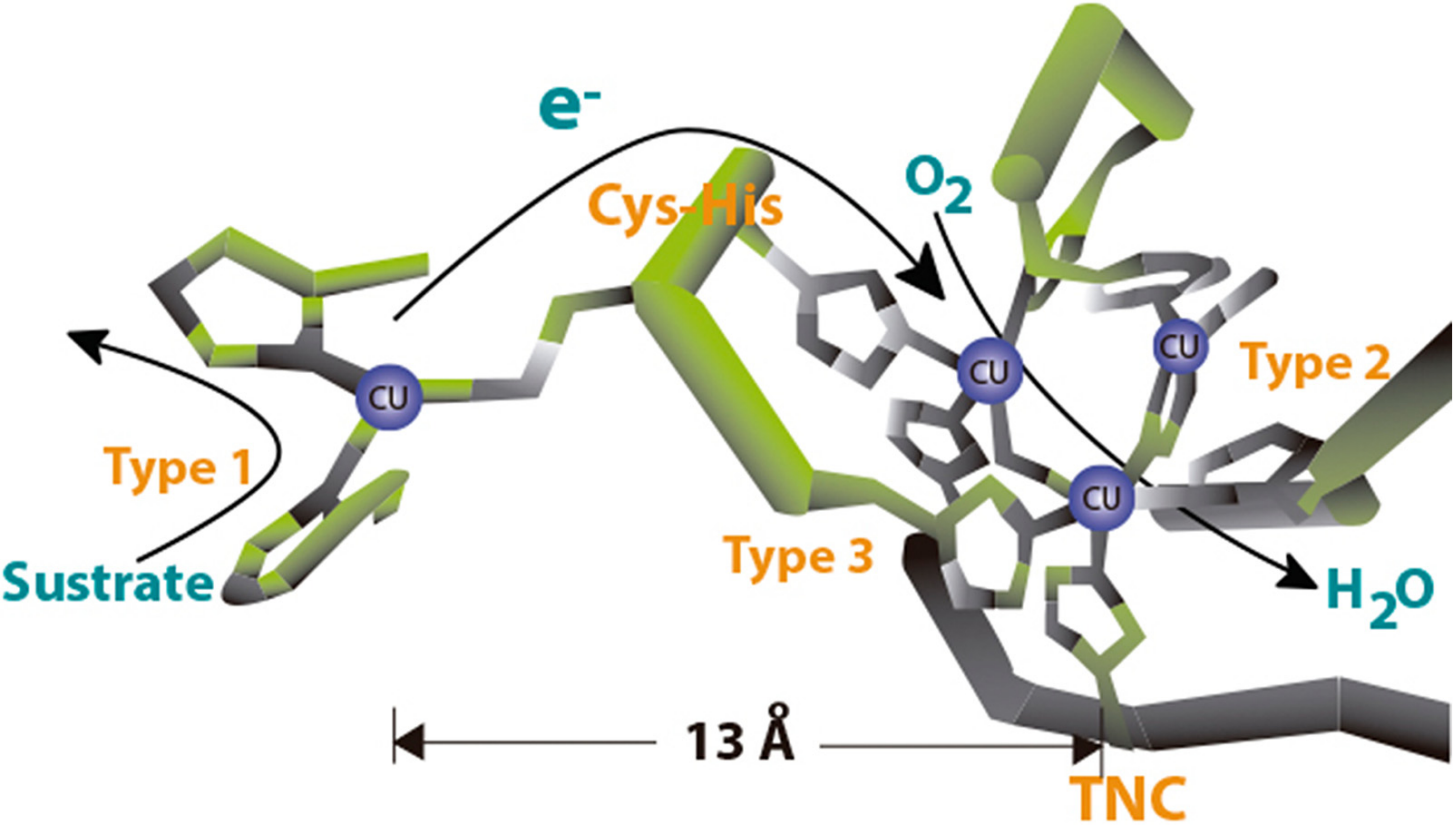
MCO active site with arrows marking electron flow from the T1 to the trinuclear site.

MCOs receive electrons at the T1 Cu site from electron donating substrates [16]. The electrons are then transferred to the T2–T3 cluster, composed of one T2 Cu and two T3 Cu ions, where O_2_ is reduced directly to water in a four-electron reduction process (Fig 2) [7].

**Fig 3:**
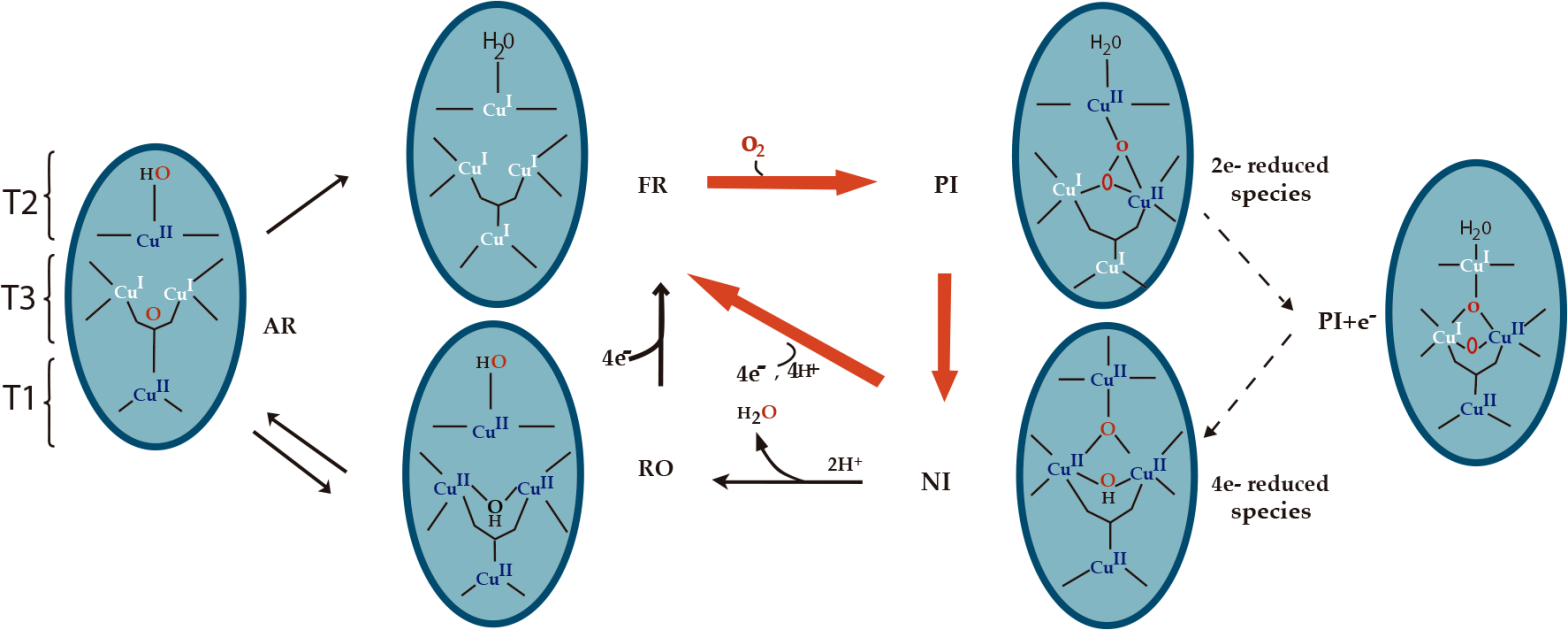
Mechanism of O_2_ reduction to water by the MCOs. Red arrows indicate fast reactions; black arrows indicate steps that are part of catalysis.

The steps involved in the reduction of oxygen to water consider that MCOs go through various oxidation states: the fully reduced enzyme (FR), the peroxy intermediate (PI), and the native intermediate (NI) (Fig 3). An overview of the redox state of the various copper atoms is presented in Fig 3.

According to a generally accepted model, the O_2_ reduction involves two-electron transfers, starting from a fully reduced enzyme [17]. In the first step, O_2_ is reduced by two electrons, forming the PI. This is followed by a second two-electron transfer, which results in cleavage of the O-O bond and formation of the NI. The catalytic cycle is completed upon reduction of NI by a total of four electrons, regenerating the FR. Alternatively, once the O-O bond has been cleaved to form NI, if the substrate is not present in excess, it will decay slowly to the fully oxidized resting form of the enzyme, the resting oxidized (RO) form, which will require reduction by the substrate for activation to return to the catalytic cycle [7,17]. The rate of conversion from NI to RO is considerably slower (k≅0.034 s-1) than the turnover rate for the enzyme (k≅560 s^-1^) [11,18], and therefore it is excluded from the catalytic cycle (in Fig 3, red arrow fast reactions, black arrows slow reactions).

**Fig 4:**
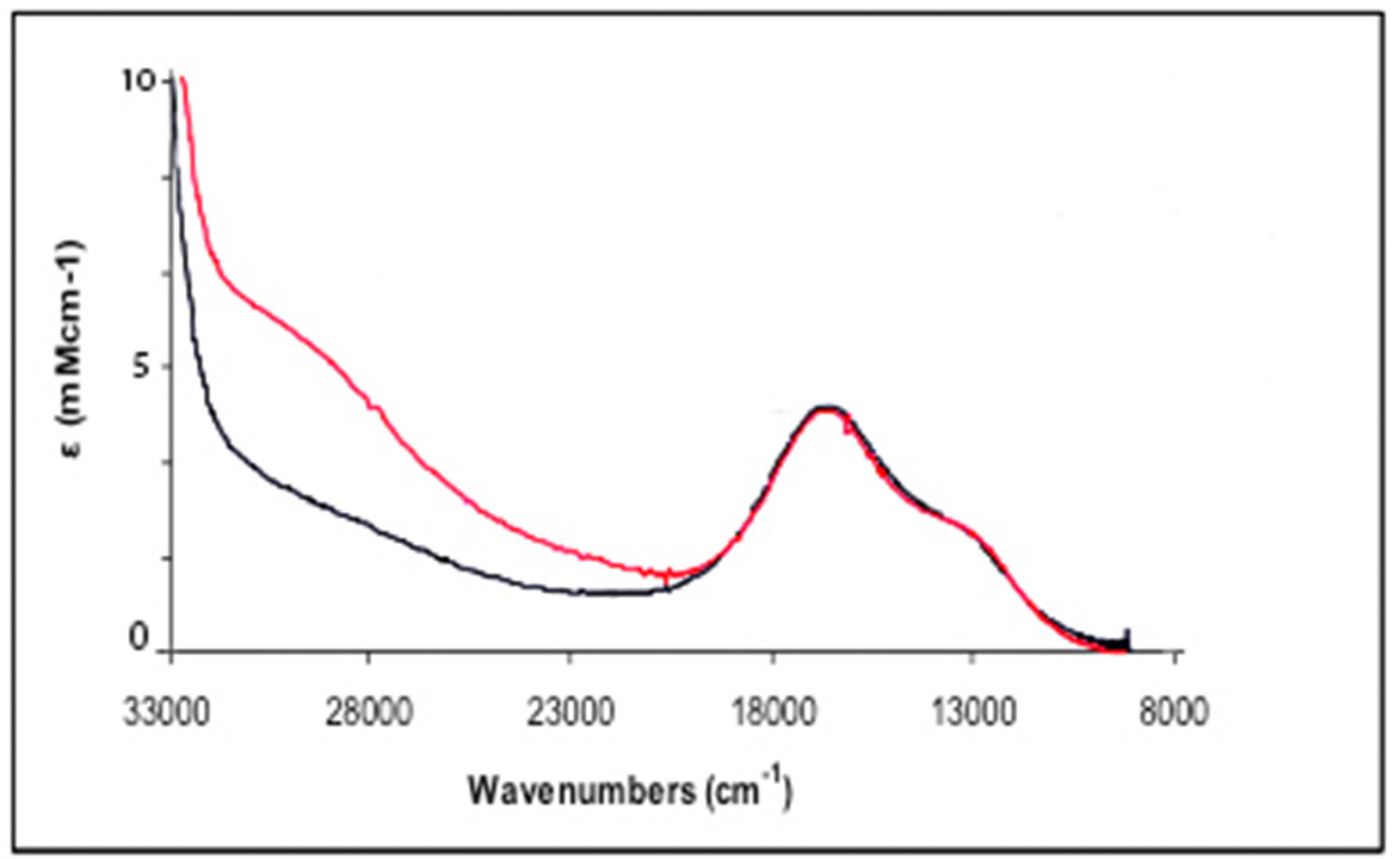
UV-visible absorption spectra of the AR form of *Mv*BOD (black) and the reoxidized form (red).

In a recent work it was found that BOD solutions prepared from *Magnaporthe oryzae* were not active [19]. They also presented a different absorption spectrum and different EPR features than the RO form M. verrucaria BOD [19]. Therefore, two interconvertible forms of resting MCOs were characterized [19], one corresponding to the previously reported RO and one indicated as alternative resting state (AR) (left side of Fig 3). The AR state presented a long T3 Cu−Cu distance, an almost planar angle between the two coppers, and a partially reduced low redox potential trinuclear site that is not suitable for O_2_ reduction. In Fig 4 we present the UV–visible absorption spectra of a prepared solution (black) and reoxidized solution of *Mv*BOD (red).

The prepared solution is spectroscopically recognized as the AR state of the enzyme, while to obtain the reoxidized state of the enzyme dithionite had to be added anaerobically to the AR form of the enzyme to allow for reduction of the T1 followed by the reduction of the trinuclear site. After reduction the enzyme solution was exposed to air and the buffer was exchanged by diafiltration, and finally the reoxidized spectrum was obtained, and it was recognized as the RO form of the enzyme. When this RO form was incubated with a NaCl solution of 0.1 M final concentration at room temperature in the presence of oxygen for a short time (<1 h) and then rebuffered in pH 7 buffer, the spectrum attributed to the AR form was again obtained.

**Fig 5:**
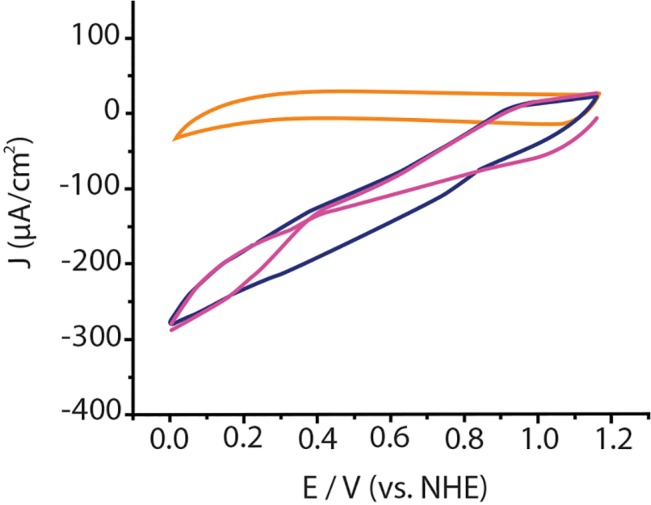
CVs of immobilized *Mv*BOD on SPGE. Orange lines in absence of O_**2.**_ Purple lines first scan; blue lines second scan. Scan rate 5 mV/s.

Fig 5 shows the cyclic voltammograms of the first (purple lines) and second (blue lines) scans of a *Mv*BOD modified electrode in air-saturated sodium phosphate buffer. Orange lines represent a cyclic voltammogram of the modified electrode in absence of O_2_. The catalytic current densities presented from the second to the subsequent scans are quite impressive and start at a redox potential of <800 mV vs. NHE, while during the first scan a catalytic onset is seen at <400 mV vs. NHE, indicating that the enzyme needs to be fully reduced before it gets activated for catalysis. This is consistent with the reduction by dithionite (low potential reductant) and the results reported in [19]. To consider the existence of an AR non-catalytically active form of the enzyme and to know how to avoid its formation is of great importance when designing biocathodes, which need to be constantly active.

Conversely, Shleev et al. have shown for *Mv*BOD-modified SPGE under anaerobic conditions a quasi-reversible peak with a mid-point potential of 515 mV at pH 7.4 [20], while in the presence of O_2_ they could observe catalysis at f 700 mV. In a subsequent study, similar experimental conditions were applied to BOD from *Trachyderma tsudonae* (TtBOD) [21]. Electrocatalytic O_2_ reduction was observed again at a similar potential (~700 mV at pH 7), but this time the authors proposed that two different orientations of the enzyme caused the electron to be transfer to different sites of the immobilized enzyme. Symmetric peaks were observed in CV conducted under anaerobic conditions, with two separate midpoint potentials of 390 and 690 mV. The peak at 690 mV was ascribed as direct electron transfer to the T1 Cu site [22]. The low-potential peak was ascribed as direct electron transfer to the T2 Cu site of the trinuclear site. In a later study, the authors immobilized BOD on gold electrodes via mercaptopropionic functional groups [21]. Under anaerobic conditions there was a single quasi-reversible peak with a mid-point potential of 360 mV; no ET at high potentials was observed, indicating limited connection to the T1 Cu site. Electrocatalytic current was seen in chronoamperometric experiments with the potential held at 500 mV. In the same study two other peaks were seen under anaerobic conditions with E° of ~390 and 690 mV for *T*. *tsunodae* BOD immobilized on an SPGE electrode. In the presence of O_2_, catalytic onset was observed at the two different potentials corresponding to the anaerobic peaks. The authors assigned it to the direct electron transfer to the trinuclear site and propose that an uphill ET from the T1 to the T2 Cu occurs in catalysis. Frasconi et al. found two redox peaks under anaerobic conditions for laccases from *T*. *versicolor* and *T*. *hirsuta* in a polymeric film of an anion exchange resin immobilized on gold electrodes [23]. Consistent with the T1 Cu potential of these enzymes, one redox peak was observed at ~800 mVand the other at ~400 mV. In contrast with the above studies, only one high potential onset was observed for catalytic conditions, consistent with the mechanism for O_2_ reduction by MCOs.

While the peaks at <700 mV can be assigned to T1 Cu reduction, consistent with the potential of this Cu determined in solution, the origin of the second peak is unclear. A low potential trinuclear site Cu may be present under anaerobic conditions, the fact that it is also seen under aerobic conditions does not fit within the context of the generally accepted model for catalytic O_2_ reduction of MCOs. In fact, under turnover conditions, the reduction of the trinuclear site of the NI is easy, ruling out the presence of a low potential trinuclear site Cu under turnover. Therefore those peaks at ~400 mV under aerobic conditions should be attributed to the AR form of the enzyme, with the low potential form converted into the high potential form when fully reduced at less than ~400 mV [19].

**Fig 6:**
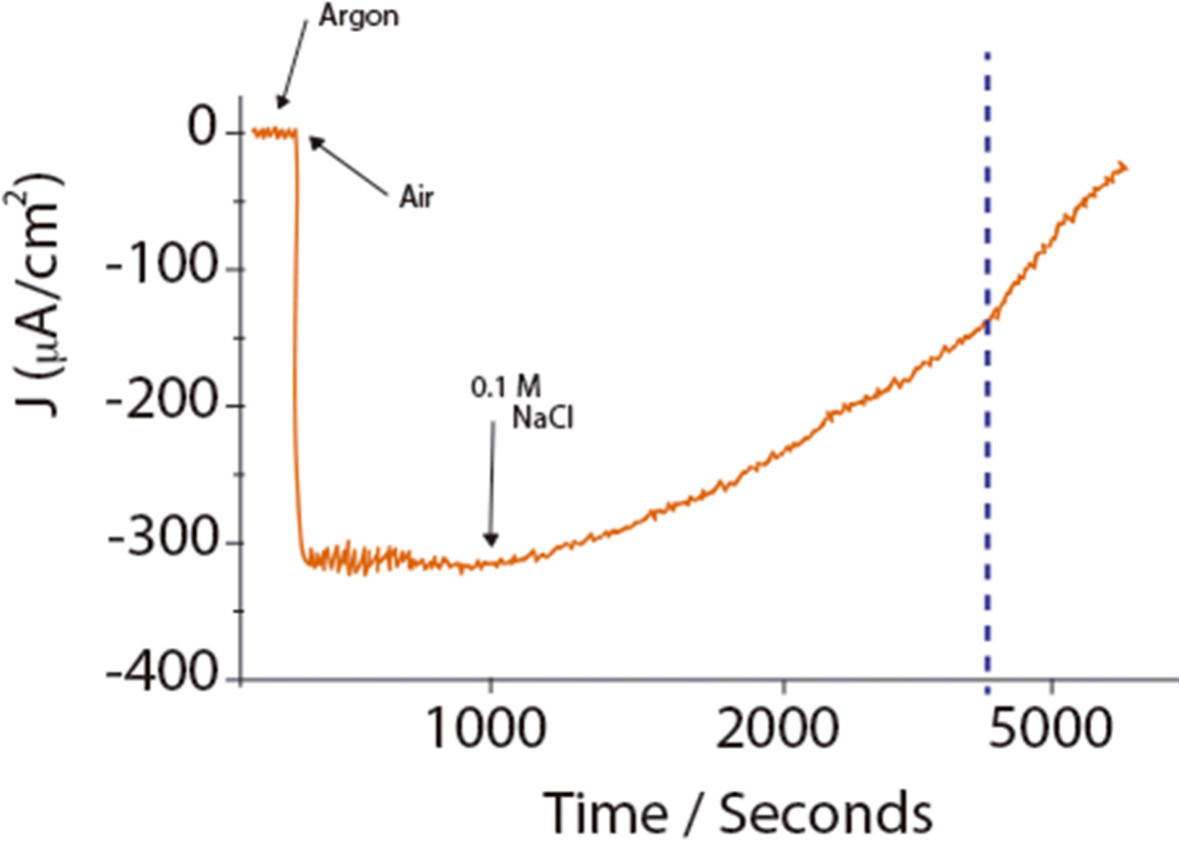
Chronoamperometric trace for *Mv*BOD on SPGE. After 1000s NaCl for a final concentration of 0.1 M was added. Sodium phosphate (0.1 M) buffer, pH 7. Electrode set at 600 mV vs. NHE.

Chloride inhibition has been debated and contrasting results have been presented [15]. If a device has to be implanted in animals (i.e., implantable biofuel cells) the presence of chloride has to be taken into account. Early studies conducted by Heller and co-workers found very weak Cl^-^ inhibition of BODs when these were immobilized on electrodes in the presence of Os-redox polymers [24–26]. For immobilized MCOs, in direct electron transfer with the electrode, different results have been obtained with respect to Cl^−^inhibition. In a study by Blanford et al., *T*.*versicolor* laccase lost more than 50% of its activity in the presence of 25 mM NaCl [27] when immobilized on an anthracene-modified graphite electrode. In contrast, Vaz-Dominguez et al. found practically no inhibition of *T*. *hirsuta* laccase, with NaCl > 350 mM [28]. Fig 6 shows chronoamperometry measurements for oxygen reduction with the electrode poisoned at 600 mV.

After approximately 16 min of chronoamperometry measurements, NaCl was added to reach a final bulk concentration of 0.1 M chloride. As seen in Fig 6, 50% of electrocatalytic current (i.e., activity) is lost in less than 1 h. If the electrode is subsequently placed in buffer without chloride, activity is not recovered. But if we reduce the enzyme at less than 400 mV poisoning the electrode at 200 mV or running a cyclic voltammetry experiment, then up to 95% of the activity is recovered. We believe that the activity loss is due to conversion of the enzyme to the AR form. However, the results obtained for electrocatalytic current inhibition with enzyme in direct electron transfer and in situations mediated via Os-complexes are ambiguous, and further tailored experiments are required for a better understanding of the impact of chloride on the catalytic activity of the enzyme [15]. Similarly to chloride, BOD appears to be inhibited also by hydrogen peroxide. In a recent study by Slade et al. it is shown that BOD loses almost 90% of the activity in the presence of 20 mM H_2_O_2_ [29]. It would be interesting to determine if the activity can be recovered after reduction of the enzyme. The evaluation of another inhibitor like fluoride in view of those new insights would also be valuable.

## Conclusions

*Mv*BOD catalyzes the ORR with higher efficiency than Pt and with almost no overpotential. Because of those characteristics, *Mv*BOD and all MCOs in general have generated significant interest during the last decade. In fact, MCOs are interesting enzymes not only for the development of biocathodes, but also because the understanding of the reaction mechanism can provide insights for the synthesis of more effective catalysts than Pt.

*Mv*BOD was physically adsorbed on spectrographite electrodes. Direct electron transfer catalytic currents were obtained in the presence of the substrate (i.e., oxygen). Catalytic activity profiles changing temperature and pH are presented. We summarize the catalytic and the non-catalytic cycle of the enzyme considering the latest published insights. We show that when the lyophilized enzyme is in solution it is present in a noncatalytic resting form called *alternative resting* (AR), which needs to be completely reduced at potentials lower than 400 mV to become catalytically active. We give new interpretations of previously reported results that did not consider the existence of the AR form of the enzyme. We report chloride inhibition for BOD when it is immobilized on SPGE without the use of redox polymers. We correlate the inhibition with the conversion to the AR form of the enzyme. All these considerations are extremely important when designing biocathodes, because conversion to AR causes loss of the catalytic activity.

## References

[1] Gewirth AA, Thorum MS. Electroreduction of Dioxygen for Fuel-Cell Applications: Materials and Challenges. Inorg Chem. 2010;49: 3557–3566. 10.1021/ic9022486 20380457

[2] Balzani V. Electron transfer in chemistry; Balzani V, Piotrowiak P, Rodgers MAJ, Mattay J, Astruc D et al, editors. Weinheim, Germany: WILEY-VCH Verlag GmbH & Co. KGaA 2001.

[3] Gray HB, Winkler JR. Electron transfer in metalloproteins In: Balzani V, editor. Electron transfer in chemistry: Wiley-VCH 2001.

[4] Ludwig R, Harreither W, Tasca F, Gorton L. Cellobiose Dehydrogenase: A Versatile Catalyst for Electrochemical Applications. Chemphyschem. 2010;11: 2674–2697. 10.1002/cphc.201000216 20661990

[5] Stone JR, Yang SP. Hydrogen peroxide: A signaling messenger. Antioxid. Redox Signal. 2006;8: 243–270. 16677071 10.1089/ars.2006.8.243

[6] Armaroli N, Balzani V. The future of energy supply: Challenges and opportunities. Angew Chem Int Ed. 2007;46: 52–66. 10.1002/anie.200602373 17103469

[7] Solomon EI, Ginsbach JW, Heppner DE, Kieber-Emmons MT, Kjaergaard CH, Smeets PJ, et al Copper dioxygen (bio) Inorg Chem. Faraday Discuss. 2011;148: 11–39. 21322475 10.1039/c005500j PMC3062954

[8] Mano N, Fernandez JL, Kim Y, Shin W, Bard AJ, Heller A. Oxygen is electroreduced to water on a "wired" enzyme electrode at a lesser overpotential than on platinum. J. Am Chem Soc. 2003;125: 15290–15291. 14664563 10.1021/ja038285d

[9] McCrory CCL, Ottenwaelder X, Stack TDP, Chidsey CED. Kinetic and mechanistic studies of the electrocatalytic reduction of O(2) to H(2)O with mononuclear Cu complexes of substituted 1,10-phenanthrolines. J Phys Chem A. 2007;111: 12641–12650. 18076134 10.1021/jp076106z

[10] Zaitseva I, Zaitsev V, Card G, Moshkov K, Bax B, Lindley P. The X-ray structure of human serum ceruloplasmin at 3.1 angstrom: Nature of the copper centres. J. Biol. Inorg Chem. 1996;1: 15–23.

[11] Solomon EI, Augustine AJ, Yoon J. O(2) Reduction to H(2)O by the multicopper oxidases. Dalton Trans. 2008: 3921–3932. 10.1039/b800799c 18648693 PMC2854021

[12] Murao S, Tanaka N. A New Enzyme “Bilirubin Oxidase” Produced by Myrothecium verrucaria MT-1. Agric Biol Chem. 1981;45: 2383–2384.

[13] Doumas BT, Perry B, Jendrzejczak B, Davis L. Measurement of direct bilirubin by use of bilirubin oxidase. Clin Chem. 1987;33: 1349–1353. 3608152

[14] Cadet M, Brilland X, Gounel S, Louerat F, Mano N. Design of a highly efficient O_2_ cathode based on bilirubin oxidase from *Magnaporthe oryzae* . ChemPhysChem Commun. 2013;14: 2097–2100. 10.1002/cphc.201300027 23401094

[15] Mano N, Edembe L. Bilirubin oxidases in bioelectrochemistry: Features and recent findings. Biosens Bioelectron. 2013;50: 478–485. 10.1016/j.bios.2013.07.014 23911663

[16] Bard AJ, Stratmann M, Rubinstein I, Fujihira M, Rusling JF. Encyclopedia of Electrochemistry, Modified Electrodes: Wiley 2007.

[17] Messerschmidt A, Ladenstein R, Huber R, Bolognesi M, Avigliano L, Petruzzelli R, et al Refined crystal-structure of ascorbate oxidase at 1.9 A resolution. J Mol Biol. 1992;224: 179–205. 1548698 10.1016/0022-2836(92)90583-6

[18] Petersen L, Degn H. Steady state kinetics of laccase from Rhus vernicifera. Biochim Biophys Acta. 1978;526: 85–92. 150864 10.1016/0005-2744(78)90292-9

[19] Kjaergaard CH, Durand F, Tasca F, Qayyum MF, Kauffmann B, Gounel S, et al Spectroscopic and Crystallographic Characterization of "Alternative Resting" and "Resting Oxidized" Enzyme Forms of Bilirubin Oxidase: Implications for Activity and Electrochemical Behavior of Multicopper Oxidases. J Am Chem Soc. 2012; 134: 5548–5551. 10.1021/ja211872j 22413777 PMC3339634

[20] Shleev S, El Kasmi A, Ruzgas T, Gorton L. Direct heterogeneous electron transfer reactions of bilirubin oxidase at a spectrographic graphite electrode. Electrochem Commun. 2004;6: 934–939.

[21] Ramirez P, Mano N, Andreu R, Ruzgas T, Heller A, Gorton L, et al Direct electron transfer from graphite and functionalized gold electrodes to T1 and T2/T3 copper centers of bilirubin oxidase. BBA-Bioenergetics. 2008;1777: 1364–1369. 10.1016/j.bbabio.2008.06.010 18639515

[22] Christenson A, Shleev S, Mano N, Heller A, Gorton L. Redox potentials of the blue copper sites of bilirubin oxidases. BBA-Bioenergetics. 2006; 1757: 1634–1641. 17020746 10.1016/j.bbabio.2006.08.008

[23] Frasconi M, Boer H, Koivula A, Mazzei F. Electrochemical evaluation of electron transfer kinetics of high and low redox potential laccases on gold electrode surface. Electrochim Acta. 2010;56: 817–827.

[24] Mano N, Kim HH, Zhang YC, Heller A. An oxygen cathode operating in a physiological solution. J Am Chem Soc. 2002;124: 6480–6486. 12033879 10.1021/ja025874v

[25] Mano N, Kim HH, Heller A. On the relationship between the characteristics of bilirubin oxidases and O-2 cathodes based on their "wiring". J Phys Chem B. 2002;106: 8842–8848.

[26] Mano N, Heller A. A miniature membraneless biofuel cell operating at 0.36 V under physiological conditions. J Electrochem Soc.2003;150: A1136–A1138.

[27] Blanford CF, Foster CE, Heath RS, Armstrong FA. Efficient electrocatalytic oxygen reduction by the 'blue' copper oxidase, laccase, directly attached to chemically modified carbons. Faraday Discuss. 2008;140: 319–335. 19213324 10.1039/b808939f

[28] Vaz-Dominguez C, Campuzano S, Rudiger O, Pita M, Gorbacheva M, Shleev S, et al Laccase electrode for direct electrocatalytic reduction of O-2 to H2O with high-operational stability and resistance to chloride inhibition. Biosens Bioelectron. 2008;24: 531–537. 10.1016/j.bios.2008.05.002 18585029

[29] Milton RD, Giroud F, Thumser AE, Minteer SD, Slade RCT. Bilirubin oxidase bioelectrocatalytic cathodes: the impact of hydrogen peroxide. Chem Commun. 2014;50: 94–96. 10.1039/c3cc47689h 24185735

